# Household and community socioeconomic and environmental determinants of child nutritional status in Cameroon

**DOI:** 10.1186/1471-2458-6-98

**Published:** 2006-04-17

**Authors:** Roland Pongou, Majid Ezzati, Joshua A Salomon

**Affiliations:** 1 Harvard School of Public Health, Boston, MA, USA; 2 Population Studies and Training Center, Brown University, Providence, RI, USA

## Abstract

**Background:**

Undernutrition is a leading cause of child mortality in developing countries, especially in sub-Saharan Africa. We examine the household and community level socioeconomic and environmental factors associated with child nutritional status in Cameroon, and changes in the effects of these factors during the 1990s economic crisis. We further consider age-specific effects of household economic status on child nutrition.

**Methods:**

Child nutritional status was measured by weight-for-age (WAZ) and height-for-age (HAZ) z-scores. Data were from Demographic and Health Surveys conducted in 1991 and 1998. We used analysis of variance to assess the bivariate association between the explanatory factors and nutritional status. Multivariate, multilevel analyses were undertaken to estimate the net effects of both household and community factors.

**Results:**

Average WAZ and HAZ declined respectively from -0.70 standard deviations (SD), i.e. 0.70 SD *below *the reference median, to -0.83 SD (p = 0.006) and from -1.03 SD to -1.14 SD (p = 0.026) between 1991 and 1998. These declines occurred mostly among boys, children over 12 months of age, and those of low socioeconomic status. Maternal education and maternal health seeking behavior were associated with better child nutrition. Household economic status had an overall positive effect that increased during the crisis, but it had little effect in children under 6 months of age. Improved household (water, sanitation and cooking fuel) and community environment had positive effects. Children living in the driest regions of the country were consistently worst off, and those in the largest cities were best off.

**Conclusion:**

Both household and community factors have significant impact on child health in Cameroon. Understanding these relationships can facilitate design of age- and community-specific intervention programs.

## Background

Childhood and maternal undernutrition is currently the single leading cause of the global burden of disease [1]. The fraction of total global health loss attributable to undernutrition was 9.5% in the year 2000, and 14.9% in high-mortality developing regions [1]. In Cameroon, the prevalence of childhood stunting and underweight rose from 23% to 29% and from 16% to 23%, respectively, between 1991 and 1998 [2], mirroring the trends in under-5-mortality rates, which increased from 126 per thousand to 152 per thousand between 1991 and 1998 [3,4]. Worsening child nutritional status in Cameroon during the 1990s was in line with highest burden of malnutrition in Africa [1], but was opposite to downward trends in much of the world [5], with many countries experiencing growth and nutrition transitions [6]. Understanding the determinants of malnutrition and their trends during periods of declining health is crucial for policy design.

Poor nutritional status reflects an imbalance in dietary intake and/or infectious diseases [7-9], and therefore is affected by multiple environmental and socioeconomic factors such as household socioeconomic status (SES), maternal education, household hygiene, and access to case management in health services [10-16]. Variation by child age in the nutritional effect of household SES and the effect of community-level infrastructure (e.g. hygiene or health service delivery) on malnutrition also have been recognized, but have been investigated in only a few studies [17-19]. Studies combining individual, household and community factors in a single analytical framework are still needed to provide reliable information for policy and program design.

Cameroon is characterized by a marked socio-cultural, economic and environmental diversity likely to cause variations in health and nutrition in children. However, few studies on determinants of child nutritional status have been conducted in Cameroon, and almost all have been in a single district, town or province [20-22]. Further, the variables included in these studies were all at the level of the household, without the inclusion of community characteristics. Therefore the effects of geographical variables and countrywide socioeconomic factors on child nutrition have not been evaluated. Key studies on nutrition and child health conducted at the country or regional level either identified the multiple determinants of health without quantifying their effects in a multivariate framework [23], or estimated the cross-level interactions of particular household and community factors [19], but with no reference to the role of important geographical variables like region of residence, an important predictor of child nutritional status [2] and a notable confounder of the SES-malnutrition relationship in the country. Different regions in Cameroon exhibit different levels of economic development as well as variation in climatic conditions and food production likely to affect child health independently of household or neighborhood economic status [24,25].

In this study, we use nationally representative household surveys from 1991 and 1998 and comparable measures across years to examine the role of multiple household, community, and regional socioeconomic and environmental variables in childhood undernutrition in Cameroon. We include an analysis of how the nutritional effect of household economic status may vary by child age. By considering data from two periods, between which there were important changes in national economy, we also consider how the effects of these determinants may change in response to macroeconomic factors.

## Methods

### Data sources

Demographic and Health Surveys (DHS) [26] were conducted in Cameroon in 1991 and 1998, designed to be representative at the national, urban-rural and regional level. A two-stage probabilistic sampling technique was used to select clusters at the first level and households at the second level (table 1). The household response rate was 83% in 1991 and 94% in 1998. The survey included a household module, as well as a questionnaire administered to women aged 15 to 49 years, comprising a birth history, information on individual characteristics and health behaviors, and details on their children.

For children alive at each survey (those under age 5 years in 1991 and under age 3 years in 1998), weight and height were measured and used to calculate anthropometric indicators. For the purpose of cross-year comparability, we restricted our study to children under 3 in both surveys. Of the 1966 children born after 1988 and surviving to 1991, anthropometric data were available for 1587 (80.7% of the weighted sample), and of the 2260 children born after 1995 and surviving to 1998, anthropometric data were reported for 1923 (85%).

### Variables

Child nutritional status is measured by weight-for-age z-scores (WAZ) and height-for-age z-scores (HAZ) using the United States National Center for Health Statistics/WHO international reference population. WAZ has been used in many epidemiological studies of undernutrition and child mortality, including in the latest systematic review and meta-analysis [27], and is suitable for the analysis of multiple determinants of child health, including socioeconomic determinants [12]. HAZ is an indicator for linear growth and reflects cumulated and chronic child health conditions. At the individual and household level (referred to as level 1), independent variables included mother's characteristics (education, health seeking behavior, age at child birth, and marital status), household variables (economic status, source of drinking water, sanitation and cooking fuel), and child characteristics (age, sex, size at birth, breastfeeding status and preceding birth interval). Individual and household variables were considered at the same level because there was less than one child per household in our data (table 1). Details on the definitions and distributions of these variables appear in table 2.

Household economic status and maternal health-seeking behavior (MHSB) are index variables constructed based on the statistical model developed by Ferguson et al. [28]. The model was designed to measure economic status based on possession of household consumer durables such as electricity, television, bicycle and car. The basic premise of the model is that wealthier households are more likely to own any given set of assets; and that some assets are likely to be consumed at relatively low levels of economic status (e.g. radio or bicycle), while others will be owned only at higher levels (e.g. television or car). The model postulates a continuous level of economic status (unobserved) predicted by a series of socio-demographic covariates (age and sex of the head of the household, mother's education and occupation, and urban or rural place of residence), with observed ownership of each asset captured in a set of indicator variables. The inclusion of certain assets presumed to be owned at roughly the same level on an internationally comparable economic status scale allows comparisons across countries or over time. A similar approach was used to estimate levels of MHSB, with predictive covariates including mother's education and occupation, and place of residence, and dichotomous indicator variables for prenatal visit, tetanus injection during pregnancy, medical assistance at delivery, knowledge of oral rehydration solutions (ORS) and possession of a health card for the child.

Increasing awareness of the effects of community or neighborhood on health beyond individual and household-level influences has produced a vast literature [29,30]. In developing countries, community-level factors that may influence health include community economic development, climatic conditions (these two factors are often captured by region of residence) and environmental hygiene. Children residing in clean neighborhoods may have better health than similar children living in unclean neighborhoods. In our study, community statistical units were the DHS clusters sample for the years 1991 and 1998, and community-level independent variables (referred to as level 2 variables) included place of residence, region of residence and environmental status (table 2). Community environmental status is a continuous variable built using pooled 1991 and 1998 datasets and principal components analysis on 5 variables including the proportion of children in households with access to water, sanitation, electricity, using electric stove or gas as cooking fuel, or having finished floor in each cluster. This variable therefore represents access to clean environmental conditions at the community level, affected by both household resources and the broader community-level infrastructure (e.g. waste disposal infrastructure and distance to water source or electricity grid).

### Statistical analysis

Analysis of variance (ANOVA) was used to assess the bivariate association between average WAZ and HAZ and selected independent variables, including child sex and age, maternal education, MHSB, economic status (the latter two recoded into 5-level variables using the quintile cut-off points of the pooled 1991 and 1998 data), water, sanitation, cooking fuel, and place and region of residence. A two tailed t-test was also performed to test the statistical significance of the change in the average WAZ and HAZ between 1991 and 1998 (table 4).

In multivariate analysis, we pooled the 1991 and 1998 data and used a two-level random intercept model to estimate the specific effect of household and community independent variables on WAZ and HAZ, using the Stata 9 statistical software. This model takes into account the hierarchical sample selection design characterizing the DHS surveys, and further adjusts for spatial correlation and heteroskedasticity, as the nutritional status of children living in the same neighborhoods (hereafter 'cluster') may be correlated due to common neighborhoods influences (e.g. access to water, electricity, etc.) This model also allows estimation of the fraction of variance of the dependent variable occurring at each level of the analysis [31,32], and is specified (for WAZ) as follows.

WAZij=β0+β1year98+∑k(βk91xkij*year91+βk98xkij*year98)+∑k(δk91zkj*year91+δk98zkj*year98)+μj+εij     (1)
 MathType@MTEF@5@5@+=feaafiart1ev1aaatCvAUfKttLearuWrP9MDH5MBPbIqV92AaeXatLxBI9gBaebbnrfifHhDYfgasaacH8akY=wiFfYdH8Gipec8Eeeu0xXdbba9frFj0=OqFfea0dXdd9vqai=hGuQ8kuc9pgc9s8qqaq=dirpe0xb9q8qiLsFr0=vr0=vr0dc8meaabaqaciaacaGaaeqabaqabeGadaaakeaacqqGxbWvcqqGbbqqcqqGAbGwdaWgaaWcbaGaemyAaKMaemOAaOgabeaakiabg2da9GGaciab=j7aInaaBaaaleaacqaIWaamaeqaaOGaey4kaSIae8NSdi2aaSbaaSqaaiabigdaXaqabaGccqWG5bqEcqWGLbqzcqWGHbqycqWGYbGCdaWgaaWcbaGaeGyoaKJaeGioaGdabeaakiabgUcaRmaaqafabaWaaeWaaeaacqWFYoGydaqhaaWcbaGaem4AaSgabaGaeGyoaKJaeGymaedaaOGaemiEaG3aaSbaaSqaaiabdUgaRjabdMgaPjabdQgaQbqabaGccqGGQaGkcqWG5bqEcqWGLbqzcqWGHbqycqWGYbGCdaWgaaWcbaGaeGyoaKJaeGymaedabeaakiabgUcaRiab=j7aInaaDaaaleaacqWGRbWAaeaacqaI5aqocqaI4aaoaaGccqWG4baEdaWgaaWcbaGaem4AaSMaemyAaKMaemOAaOgabeaakiabcQcaQiabdMha5jabdwgaLjabdggaHjabdkhaYnaaBaaaleaacqaI5aqocqaI4aaoaeqaaaGccaGLOaGaayzkaaaaleaacqWGRbWAaeqaniabggHiLdGccqGHRaWkdaaeqbqaamaabmaabaGae8hTdq2aa0baaSqaaiabdUgaRbqaaiabiMda5iabigdaXaaakiabdQha6naaBaaaleaacqWGRbWAcqWGQbGAaeqaaOGaeiOkaOIaemyEaKNaemyzauMaemyyaeMaemOCai3aaSbaaSqaaiabiMda5iabigdaXaqabaGccqGHRaWkcqWF0oazdaqhaaWcbaGaem4AaSgabaGaeGyoaKJaeGioaGdaaOGaemOEaO3aaSbaaSqaaiabdUgaRjabdQgaQbqabaGccqGGQaGkcqWG5bqEcqWGLbqzcqWGHbqycqWGYbGCdaWgaaWcbaGaeGyoaKJaeGioaGdabeaaaOGaayjkaiaawMcaaiabgUcaRiab=X7aTnaaBaaaleaacqWGQbGAaeqaaOGaey4kaSIae8xTdu2aaSbaaSqaaiabdMgaPjabdQgaQbqabaaabaGaem4AaSgabeqdcqGHris5aOGaaCzcaiaaxMaadaqadaqaaiabigdaXaGaayjkaiaawMcaaaaa@A887@

where

WAZ_*ij *_= weight-for-age z-score for a child *i *in cluster *j*

*β*_0 _= intercept

*year*_*t *_= dummy indicator for the year *t *(*t *= 1991, 1998)

*β*_1 _= coefficient on the year 1998

*x*_*kij *_= value of variable *x*_*k *_for a child *i *in cluster *j *(*x*_*k *_is an individual or household level variable)

*x*_kij _* *year*_*t *_= interaction term between variable *x*_*k *_and year *t *(*t *= 1991, 1998) evaluated for a child *i *in cluster *j*

βkt
 MathType@MTEF@5@5@+=feaafiart1ev1aaatCvAUfKttLearuWrP9MDH5MBPbIqV92AaeXatLxBI9gBaebbnrfifHhDYfgasaacH8akY=wiFfYdH8Gipec8Eeeu0xXdbba9frFj0=OqFfea0dXdd9vqai=hGuQ8kuc9pgc9s8qqaq=dirpe0xb9q8qiLsFr0=vr0=vr0dc8meaabaqaciaacaGaaeqabaqabeGadaaakeaaiiGacqWFYoGydaqhaaWcbaGaem4AaSgabaGaemiDaqhaaaaa@3151@ = coefficient on variable *x*_*k *_in period *t *(*t *= 1991, 1998), representing increase in WAZ due to a unit increase in *x*_*k *_if *x*_*k *_is continuous, or the differential effect of *x*_*k *_on WAZ relative to a reference category if *x*_*k *_is a dummy indicator representing a category of a categorical variable

*z*_*kj *_= value of variable *z*_*k *_for a cluster *j *(*z*_*k *_is a community level variable)

*z*_kj _* *year*_*t *_= interaction term between variable *z*_*k *_and year *t *(*t *= 1991, 1998) evaluated for a cluster *j*

δkt
 MathType@MTEF@5@5@+=feaafiart1ev1aaatCvAUfKttLearuWrP9MDH5MBPbIqV92AaeXatLxBI9gBaebbnrfifHhDYfgasaacH8akY=wiFfYdH8Gipec8Eeeu0xXdbba9frFj0=OqFfea0dXdd9vqai=hGuQ8kuc9pgc9s8qqaq=dirpe0xb9q8qiLsFr0=vr0=vr0dc8meaabaqaciaacaGaaeqabaqabeGadaaakeaaiiGacqWF0oazdaqhaaWcbaGaem4AaSgabaGaemiDaqhaaaaa@3155@ = coefficient on variable *z*_*k *_in period *t *(*t *= 1991, 1998), interpreted similarly as βkt
 MathType@MTEF@5@5@+=feaafiart1ev1aaatCvAUfKttLearuWrP9MDH5MBPbIqV92AaeXatLxBI9gBaebbnrfifHhDYfgasaacH8akY=wiFfYdH8Gipec8Eeeu0xXdbba9frFj0=OqFfea0dXdd9vqai=hGuQ8kuc9pgc9s8qqaq=dirpe0xb9q8qiLsFr0=vr0=vr0dc8meaabaqaciaacaGaaeqabaqabeGadaaakeaaiiGacqWFYoGydaqhaaWcbaGaem4AaSgabaGaemiDaqhaaaaa@3151@

*μ*_*j *_= clusters residuals assumed to be independent and normally distributed

*ε*_*ij *_= within-cluster individuals residuals assumed to be independent and normally distributed.

The residual terms *μ*_*j *_and *ε*_*ij *_are assumed to have zero mean, with respective variances σμ2
 MathType@MTEF@5@5@+=feaafiart1ev1aaatCvAUfKttLearuWrP9MDH5MBPbIqV92AaeXatLxBI9gBaebbnrfifHhDYfgasaacH8akY=wiFfYdH8Gipec8Eeeu0xXdbba9frFj0=OqFfea0dXdd9vqai=hGuQ8kuc9pgc9s8qqaq=dirpe0xb9q8qiLsFr0=vr0=vr0dc8meaabaqaciaacaGaaeqabaqabeGadaaakeaaiiGacqWFdpWCdaqhaaWcbaGae8hVd0gabaGaeGOmaidaaaaa@3146@ and σε2
 MathType@MTEF@5@5@+=feaafiart1ev1aaatCvAUfKttLearuWrP9MDH5MBPbIqV92AaeXatLxBI9gBaebbnrfifHhDYfgasaacH8akY=wiFfYdH8Gipec8Eeeu0xXdbba9frFj0=OqFfea0dXdd9vqai=hGuQ8kuc9pgc9s8qqaq=dirpe0xb9q8qiLsFr0=vr0=vr0dc8meaabaqaciaacaGaaeqabaqabeGadaaakeaaiiGacqWFdpWCdaqhaaWcbaGae8xTdugabaGaeGOmaidaaaaa@3137@, which are the variance of WAZ occurring at the cluster and individual level, respectively, after netting out the effects of independent variables. Only coefficients βk91
 MathType@MTEF@5@5@+=feaafiart1ev1aaatCvAUfKttLearuWrP9MDH5MBPbIqV92AaeXatLxBI9gBaebbnrfifHhDYfgasaacH8akY=wiFfYdH8Gipec8Eeeu0xXdbba9frFj0=OqFfea0dXdd9vqai=hGuQ8kuc9pgc9s8qqaq=dirpe0xb9q8qiLsFr0=vr0=vr0dc8meaabaqaciaacaGaaeqabaqabeGadaaakeaaiiGacqWFYoGydaqhaaWcbaGaem4AaSgabaGaeGyoaKJaeGymaedaaaaa@31D0@ and βk98
 MathType@MTEF@5@5@+=feaafiart1ev1aaatCvAUfKttLearuWrP9MDH5MBPbIqV92AaeXatLxBI9gBaebbnrfifHhDYfgasaacH8akY=wiFfYdH8Gipec8Eeeu0xXdbba9frFj0=OqFfea0dXdd9vqai=hGuQ8kuc9pgc9s8qqaq=dirpe0xb9q8qiLsFr0=vr0=vr0dc8meaabaqaciaacaGaaeqabaqabeGadaaakeaaiiGacqWFYoGydaqhaaWcbaGaem4AaSgabaGaeGyoaKJaeGioaGdaaaaa@31DE@ estimating simultaneously the effects of these independent variables for the years 1991 and 1998, respectively, were reported in addition to the individual and cluster level variances σε2
 MathType@MTEF@5@5@+=feaafiart1ev1aaatCvAUfKttLearuWrP9MDH5MBPbIqV92AaeXatLxBI9gBaebbnrfifHhDYfgasaacH8akY=wiFfYdH8Gipec8Eeeu0xXdbba9frFj0=OqFfea0dXdd9vqai=hGuQ8kuc9pgc9s8qqaq=dirpe0xb9q8qiLsFr0=vr0=vr0dc8meaabaqaciaacaGaaeqabaqabeGadaaakeaaiiGacqWFdpWCdaqhaaWcbaGae8xTdugabaGaeGOmaidaaaaa@3137@ and σμ2
 MathType@MTEF@5@5@+=feaafiart1ev1aaatCvAUfKttLearuWrP9MDH5MBPbIqV92AaeXatLxBI9gBaebbnrfifHhDYfgasaacH8akY=wiFfYdH8Gipec8Eeeu0xXdbba9frFj0=OqFfea0dXdd9vqai=hGuQ8kuc9pgc9s8qqaq=dirpe0xb9q8qiLsFr0=vr0=vr0dc8meaabaqaciaacaGaaeqabaqabeGadaaakeaaiiGacqWFdpWCdaqhaaWcbaGae8hVd0gabaGaeGOmaidaaaaa@3146@ (table 5).

To estimate changes across years in the effects of independent variables, we used the following equation:

WAZij=α0+α1year98+∑k(αk98−91xkij*year98+αk91xkij)+∑k(ϕk98−91zkj*year98+ϕk91zkj)+μj+εij     (2)
 MathType@MTEF@5@5@+=feaafiart1ev1aaatCvAUfKttLearuWrP9MDH5MBPbIqV92AaeXatLxBI9gBaebbnrfifHhDYfgasaacH8akY=wiFfYdH8Gipec8Eeeu0xXdbba9frFj0=OqFfea0dXdd9vqai=hGuQ8kuc9pgc9s8qqaq=dirpe0xb9q8qiLsFr0=vr0=vr0dc8meaabaqaciaacaGaaeqabaqabeGadaaakeaacqqGxbWvcqqGbbqqcqqGAbGwdaWgaaWcbaGaemyAaKMaemOAaOgabeaakiabg2da9GGaciab=f7aHnaaBaaaleaacqaIWaamaeqaaOGaey4kaSIae8xSde2aaSbaaSqaaiabigdaXaqabaGccqWG5bqEcqWGLbqzcqWGHbqycqWGYbGCdaWgaaWcbaGaeGyoaKJaeGioaGdabeaakiabgUcaRmaaqafabaWaaeWaaeaacqWFXoqydaqhaaWcbaGaem4AaSgabaGaeGyoaKJaeGioaGJaeyOeI0IaeGyoaKJaeGymaedaaOGaemiEaG3aaSbaaSqaaiabdUgaRjabdMgaPjabdQgaQbqabaGccqGGQaGkcqWG5bqEcqWGLbqzcqWGHbqycqWGYbGCdaWgaaWcbaGaeGyoaKJaeGioaGdabeaakiabgUcaRiab=f7aHnaaDaaaleaacqWGRbWAaeaacqaI5aqocqaIXaqmaaGccqWG4baEdaWgaaWcbaGaem4AaSMaemyAaKMaemOAaOgabeaaaOGaayjkaiaawMcaaaWcbaGaem4AaSgabeqdcqGHris5aOGaey4kaSYaaabuaeaadaqadaqaaiab=v9aQnaaDaaaleaacqWGRbWAaeaacqaI5aqocqaI4aaocqGHsislcqaI5aqocqaIXaqmaaGccqWG6bGEdaWgaaWcbaGaem4AaSMaemOAaOgabeaakiabcQcaQiabdMha5jabdwgaLjabdggaHjabdkhaYnaaBaaaleaacqaI5aqocqaI4aaoaeqaaOGaey4kaSIae8x1dO2aa0baaSqaaiabdUgaRbqaaiabiMda5iabigdaXaaakiabdQha6naaBaaaleaacqWGRbWAcqWGQbGAaeqaaaGccaGLOaGaayzkaaGaey4kaSIae8hVd02aaSbaaSqaaiabdQgaQbqabaGccqGHRaWkcqWF1oqzdaWgaaWcbaGaemyAaKMaemOAaOgabeaaaeaacqWGRbWAaeqaniabggHiLdGccaWLjaGaaCzcamaabmaabaGaeGOmaidacaGLOaGaayzkaaaaaa@9D79@

Note that equation (2) is algebraically equivalent to equation (1) in that αk91
 MathType@MTEF@5@5@+=feaafiart1ev1aaatCvAUfKttLearuWrP9MDH5MBPbIqV92AaeXatLxBI9gBaebbnrfifHhDYfgasaacH8akY=wiFfYdH8Gipec8Eeeu0xXdbba9frFj0=OqFfea0dXdd9vqai=hGuQ8kuc9pgc9s8qqaq=dirpe0xb9q8qiLsFr0=vr0=vr0dc8meaabaqaciaacaGaaeqabaqabeGadaaakeaaiiGacqWFXoqydaqhaaWcbaGaem4AaSgabaGaeGyoaKJaeGymaedaaaaa@31CE@ = βk91
 MathType@MTEF@5@5@+=feaafiart1ev1aaatCvAUfKttLearuWrP9MDH5MBPbIqV92AaeXatLxBI9gBaebbnrfifHhDYfgasaacH8akY=wiFfYdH8Gipec8Eeeu0xXdbba9frFj0=OqFfea0dXdd9vqai=hGuQ8kuc9pgc9s8qqaq=dirpe0xb9q8qiLsFr0=vr0=vr0dc8meaabaqaciaacaGaaeqabaqabeGadaaakeaaiiGacqWFYoGydaqhaaWcbaGaem4AaSgabaGaeGyoaKJaeGymaedaaaaa@31D0@, ϕk91=δk91,αk98−91=βk98−βk91
 MathType@MTEF@5@5@+=feaafiart1ev1aaatCvAUfKttLearuWrP9MDH5MBPbIqV92AaeXatLxBI9gBaebbnrfifHhDYfgasaacH8akY=wiFfYdH8Gipec8Eeeu0xXdbba9frFj0=OqFfea0dXdd9vqai=hGuQ8kuc9pgc9s8qqaq=dirpe0xb9q8qiLsFr0=vr0=vr0dc8meaabaqaciaacaGaaeqabaqabeGadaaakeaaiiGacqWFvpGAdaqhaaWcbaGaem4AaSgabaGaeGyoaKJaeGymaedaaOGaeyypa0Jae8hTdq2aa0baaSqaaiabdUgaRbqaaiabiMda5iabigdaXaaakiabcYcaSiab=f7aHnaaDaaaleaacqWGRbWAaeaacqaI5aqocqaI4aaocqGHsislcqaI5aqocqaIXaqmaaGccqGH9aqpcqWFYoGydaqhaaWcbaGaem4AaSgabaGaeGyoaKJaeGioaGdaaOGaeyOeI0Iae8NSdi2aa0baaSqaaiabdUgaRbqaaiabiMda5iabigdaXaaaaaa@4D57@ and ϕk98−91=δk98−δk91
 MathType@MTEF@5@5@+=feaafiart1ev1aaatCvAUfKttLearuWrP9MDH5MBPbIqV92AaeXatLxBI9gBaebbnrfifHhDYfgasaacH8akY=wiFfYdH8Gipec8Eeeu0xXdbba9frFj0=OqFfea0dXdd9vqai=hGuQ8kuc9pgc9s8qqaq=dirpe0xb9q8qiLsFr0=vr0=vr0dc8meaabaqaciaacaGaaeqabaqabeGadaaakeaaiiGacqWFvpGAdaqhaaWcbaGaem4AaSgabaGaeGyoaKJaeGioaGJaeyOeI0IaeGyoaKJaeGymaedaaOGaeyypa0Jae8hTdq2aa0baaSqaaiabdUgaRbqaaiabiMda5iabiIda4aaakiabgkHiTiab=r7aKnaaDaaaleaacqWGRbWAaeaacqaI5aqocqaIXaqmaaaaaa@4133@. Therefore only coefficients  and ϕk98−91
 MathType@MTEF@5@5@+=feaafiart1ev1aaatCvAUfKttLearuWrP9MDH5MBPbIqV92AaeXatLxBI9gBaebbnrfifHhDYfgasaacH8akY=wiFfYdH8Gipec8Eeeu0xXdbba9frFj0=OqFfea0dXdd9vqai=hGuQ8kuc9pgc9s8qqaq=dirpe0xb9q8qiLsFr0=vr0=vr0dc8meaabaqaciaacaGaaeqabaqabeGadaaakeaaiiGacqWFvpGAdaqhaaWcbaGaem4AaSgabaGaeGyoaKJaeGioaGJaeyOeI0IaeGyoaKJaeGymaedaaaaa@34E6@ estimating respectively the cross-year changes in the effects of individual/household and community independent variables were reported (table 7). Equation (1) and equation (2) were also estimated for height-for-age z-score by replacing WAZ by HAZ (tables 6–7).

Summarizing what precedes, while equation (1) estimates the effects of independent variables for the years 1991 and 1998 simultaneously, equation (2) estimates changes in the effects of these variables between the two years.

The testing strategy used in the multivariate analysis estimated first the effects of some socioeconomic factors (tables 5–6, models 1–2) and environmental factors (tables 5–6, model 3) on nutritional status in separate equations; because maternal education and place of residence were used as predictors in the construction of economic status and MHSB, the adjusted effects of these variables were estimated separately (tables 5–6, models 4–6). We also estimated the full model including all independent variables (tables 5–6, model 7). Finally, we estimated the age-specific effects of household economic status to check variations in these effects by child age (table 8). Multilevel analyses were not weighted.

## Results

### Descriptive and bivariate analysis

Average WAZ and HAZ in children younger than 3 years old in Cameroon declined respectively from -0.70 standard deviations (SD), i.e. 0.70 SD below the reference median value, to -0.83 SD (p = 0.006), and from -1.03 SD to -1.14 SD (p = 0.026) between 1991 and 1998. During this period, the prevalence of underweight (defined as WAZ<-2 SD) and stunting (% HAZ<-2 SD) increased respectively from 16% to 22% (P < 0.0001), and from 23% to 29% (p < 0.0001). Average weight-for-height z-score (WHZ) also deteriorated, mirroring increase in the prevalence of wasting (%WHZ<-2 SD) (table 3). Because of a shift of the sample toward rural children during this period (see tables 1 and 2), trends in nutritional indicators were adjusted for place of residence and child sex and age, and we still found evidence of a decline in nutritional status. It should be noted that this sample shift might reflect a massive urban-to-rural migration flow during the 1990s economic crisis [33-35], in addition to a lower fertility decline in rural areas compared to urban areas during this period [36,37].

Results for bivariate analyses are presented in table 4. The decline in nutritional status occurred mostly in boys, children aged 12–23 months, those born to uneducated mothers, and those of low economic status. Child sex was not significantly associated with nutritional status in 1991, but girls had higher average WAZ and HAZ compared to boys in 1998 due to uneven declines. Child age was also a significant predictor of nutritional status.

**Figure 1a-b:**
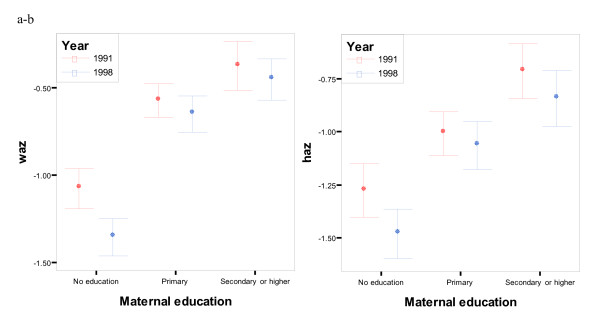
Educational gradient in weight-for-age (WAZ) and height-for-age z-scores (HAZ) in 1991 and 1998 in Cameroon. error bars represent 95% CI around the mean.

**Figure 2a-b:**
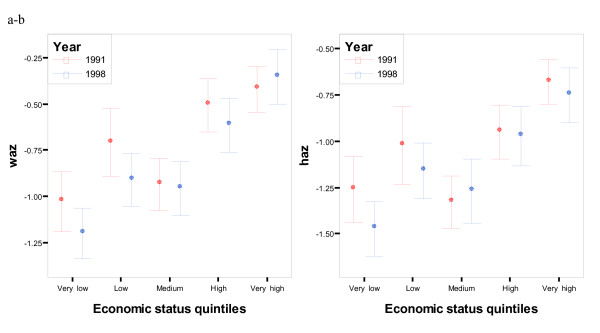
Effect of household economic status on WAZ and HAZ. error bars represent 95% CI around the mean.

**Figure 3a-b:**
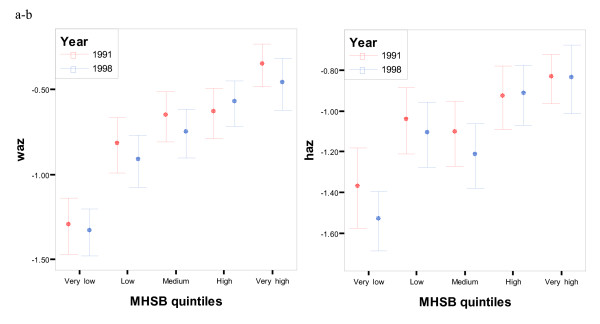
Effect of MHSB on WAZ and HAZ. error bars represent 95% CI around the mean.

In both years, nutritional status improved with maternal education (table 4, figures 1a-b). Further, the advantage associated with education increased between 1991 and 1998, as decline in WAZ and HAZ was concentrated in children of uneducated mothers, although this advantage was not statistically significant when clustering of observations was taken into account (see unadjusted model in table 7). A positive economic status gradient was also noted in 1991 and 1998 (table 4, figures 2a-b). Moreover, the gap between the richest and the poorest economic groups increased during this period, although not significantly. Maternal health seeking behavior also had a significantly positive effect on nutritional status in both years (table 4, figures 3a-b). Further, the difference in HAZ between children born to mothers with the highest MHSB and those born to mothers with the lowest MHSB increased during the crisis, but not significantly.

**Figure 4a-b:**
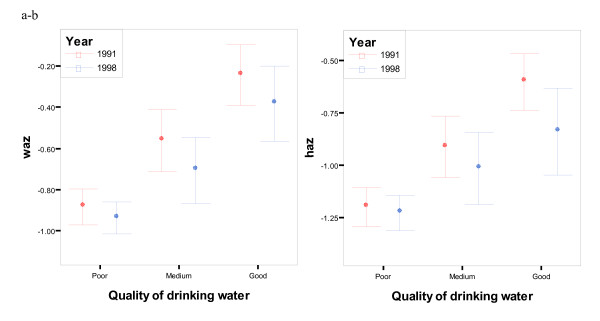
Variation in WAZ and HAZ by quality of drinking water. error bars represent 95% CI around the mean.

Improved water and cleaner cooking fuels were also associated with better nutritional status in 1991 and 1998 (table 4; also see figures 4a-b for water). Similar results were found for sanitation. Children with flush toilets in their houses had lower nutritional status than those with improved pit latrines, but this anomaly was not statistically significant.

**Figure 5a-b:**
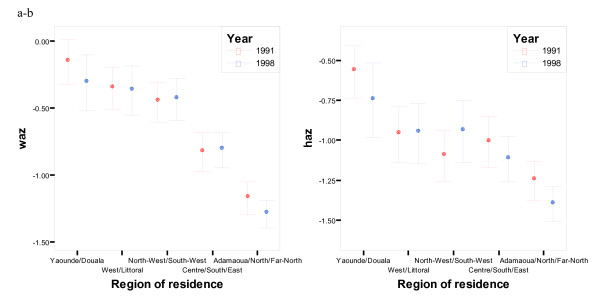
Regional disparities in WAZ and HAZ. error bars represent 95% CI around the mean.

At the community level, child nutritional status was higher in urban areas in each year (table 4). Average WAZ was -0.16 SD in the largest cities Yaounde and Douala and -0.82 SD in rural areas in 1991 (p < 0.001); it was respectively -0.31 SD and -0.91 SD in 1998 (p < 0.001). Average HAZ was -0.57 SD in Yaounde/Douala and -1.15 SD in rural areas in 1991 (p < 0.001), and was respectively -0.75 SD and -1.22 SD in 1998 (p < 0.001). In both years, the advantage of children in intermediate cities versus rural areas was not statistically significant for either nutritional indicator. Significant regional variation in child nutrition was also observed in the country (table 4; also see figures 5a-b). Children living in the West or Littoral province had the highest nutritional status after those living in the largest cities; in contrast, children in northern Cameroon had the lowest average WAZ and HAZ.

### Multivariate results

#### Effects of socioeconomic and environmental factors

The multivariate, multilevel linear regression confirmed many of the results found in bivariate analyses, but the effects of some variables declined. Controlling for economic status and MHSB showed positive effects of these variables on WAZ (table 5, model 1) and HAZ (table 6, model 1); however, the effect of MHSB completely disappeared in 1998 after additional control for maternal education, while remaining significantly positive in 1991 (table 5–6, model 2). Maternal education had a positive effect in both years. Model 3 shows positive effects of water, sanitation and cooking fuel, but some of these effects diminished in favor of community environmental status after additional control for this variable (model not shown).

Because maternal education and place of residence were used as predictors in the construction of economic status and MHSB, and because of the high correlation of economic status with community environmental status (R^2 ^= 0.74 in each year), showing that poorer households most often live in unclean neighborhoods, we estimated the effects of these variables in separate regressions after adjusting for all other variables. The effects of economic status and MHSB were still positive (tables 5–6, model 4), but the effect of economic status diminished in 1991 in favor of water, sanitation and cooking fuel after additional adjustment for these variables (tables 5–6, model 5). Also, maternal education and community environmental status still had positive effects (tables 5–6, model 6).

The full model (model 7) did not significantly change the effects of our main exposures on WAZ and HAZ. We note that either economic status or household or community environmental variables became non-significant, showing that environmental conditions are systematic modulators of the income-nutrition relationship. We also note that in general, either maternal education or MHSB became non-significant, thus highlighting the positive correlation of education with better use of primary health care facilities, and their joint positive impact on child nutrition.

The full model also showed that children living in the largest cities were still better off than those in rural areas (this is true for WAZ in both years and for HAZ in 1998), but those in the intermediate cities were eventually worse off as compared with rural areas (this is true for WAZ). This implies that the relative advantage associated with the intermediate cities over the rural areas was entirely due to the socioeconomic composition of those milieus. Regional differentials in child nutritional status remained robust to all controls for WAZ, and the relative advantage of some southern regions over the northern region increased over time, although not significantly.

#### Changes in the effects of independent variables during the crisis

We estimated changes over time in the effects of independent variables (table 7). The full model including all independent variables was first used for this purpose, but to avoid possible bias due to correlation between some variables as previously discussed, model 5 was used for all variables except for maternal education, place and region of residence, and community environmental index which were estimated from model 6. However the results of this exercise were very similar. We note that the positive effect of economic status (significant for WAZ and non-significant for HAZ) increased during the crisis, while the effects of environmental variables diminished in general. Maternal education also had a non-significant increasing effect during this period. The relative advantage of girls over boys also increased over time, while the advantage of living monogamously diminished, perhaps suggesting that the crisis had a greater impact on stable families.

#### Age-specific effects of household economic status

We estimated variation across child age groups in the nutritional effects of household economic status (table 8). We note that this variable had little effect in children under 6 months of age, but its effect was positive on length-for-age in that age group in 1998. The effect of economic status was in general significantly greater in children above 6 months, compared to younger children. This result suggests that children under 6 months of age should be distinguished from older children in the analysis of factors associated with child nutrition outcomes.

## Discussion

We examined the household and community level socioeconomic and environmental factors associated with nutritional status among children under 3 years old in Cameroon and assessed the changes in the effects of those factors between 1991 and 1998, which was a period of severe economic crisis in the country. Real GDP per capita fell from I$ 2266 in 1990 to I$ 1949 in 1998 (1996 constant price) [38]. Average weight-for-age z-score and height-for-age z-score declined respectively from -0.70 SD to -0.83 SD (p = 0.006) and from -1.03 SD to -1.14 SD (p = 0.026) during this period. The situation experienced in Cameroon during this period was opposite to global trends in malnutrition [5,39]. The prevalence of stunting declined in Africa, Asia, Latin America and the Caribbean during the 1990s, but remained stable in Western and Eastern Africa, as well as Central America. Downward trends in malnutrition were also noted in Indonesia despite the 1997/1998 financial crisis [12,40]. Growth experienced by many developing countries was followed by nutrition transition, often implying a rising pattern of obesity [6,41] or a double burden of malnutrition and obesity in some households [42].

The positive effect of maternal education and health seeking behavior on child nutritional status found in our study is consistent with other studies on factors affecting child health, such as those in India and Mali [43,44].

Economic status had a positive effect in general, but it had little effect in children aged 0–5 months, and had significantly positive effect in older ages. It is possible that the little effect of economic status in 0–5 months is due to the role of breastfeeding, which is less frequent in high-economic status mothers than lower economic status mothers due to time budget and the ability to pay for supplementation foods. The effect of economic status in children aged 0–5 months increased between 1991 and 1998 (this is particularly true for HAZ where it was positive in 1998), however, perhaps reflecting the high sensitization toward breastfeeding during the 1990s, shifting even high-economic status mothers to frequently breastfeeding their children. Unavailability of adequate data on quality of breastfeeding did not allow us to test this hypothesis. When supplementation food becomes important (after the age of 6 months), economic status is associated with improved child nutritional status because of the relative facility of high economic status mothers to afford supplementation. The age-specific effect of economic status found in our study is consistent with Sahn et al. [17], although this study used more aggregated age groups.

The positive nutritional effect of improved water and sanitation found in this study is consistent with other studies conducted in developing countries [11,45]. Unclean water may affect nutritional status through diarrhoeal diseases [46-49]. Further, we found that cleaner fuels were associated with better anthropometric indicators, consistent with the only other available study, in South India [43]. The role of clean fuels may be mediated by effects on birth weight [50], or on the risk of respiratory infections [51,52], which may in turn influence growth. Consistently with Fotso et al. [19], we also found that better community environmental status positively affected nutritional status after other factors were adjusted, suggesting that community hygiene affects health irrespectively of individual or household characteristics.

Our study suggests that urban-rural differentials in child nutritional status in Cameroon –especially those between the intermediate cities and rural areas–are mediated by the socioeconomic composition of those areas. The relative advantage of the largest cities over the rural areas declined after all controls, but remained positive (except for HAZ in 1991), which may be attributable to some contextual factors such as better access to and quality of health care in the main cities. This is consistent to some extent with Kuate [53] who found urban advantage in survival of children under 5 years old in Cameroon to be completely mediated by hospital delivery.

Our study also reports regional disparities in child nutritional status that were robust to all controls for WAZ, but that almost disappeared for HAZ, suggesting that much of the regional gap in HAZ is mediated by differential socioeconomic conditions. Northern Cameroon, which is a region with dry climate, limited food crops and limited access to health care, consistently had the worst WAZ. The Center/South/East region had the closest outcome to the North. Such a situation may prevail because of overwhelmingly low access to food and health care in the East province, which should be distinguished from the South and Center provinces which have better access to varied food and health services [53]. Disaggregating analysis to draw this distinction was not possible, due to non-representativeness of sample sizes at the province level.

There are some limitations in the data used for this analysis. Since this work relied on survey-based data, selection and recall bias could affect the results. Using a measured anthropometric indicator of nutritional outcome, however, eliminates one of the most important sources of bias. The measure of community environmental status used in this study is only a proxy of what the real environmental conditions and infrastructure are, as factors such as water stagnation, garbage accumulation and distance to electricity grid, which capture the quality of environment, were not available in our data. The availability of those indicators may have produced a better estimate of the effect of environmental infrastructure on child nutritional health. Other limitations of this work stem from the amount of missing data. Weight-for-age and height-for-age z-scores were calculated for only 81% and 85% of children under 3 years old in 1991 and 1998 respectively. Although the fraction of observations with missing value was relatively low, we found missing data to be correlated with some factors including household economic status and hygiene, MHSB, child age, and place and region of residence, a potential source of bias.

## Conclusion

The age-specific effect of economic status on child nutritional status found in this study suggests that children aged 0–5 months should be distinguished from older children in the analysis of factors associated with child nutrition outcomes, with an attention to the possible role of breastfeeding in early ages, as it has been proven to have great benefits to child health and survival [54-56]. Socioeconomic and environmental effects may vary across age groups, motivating age-specific analysis of factors affecting health. This study shows that child nutritional health is simultaneously influenced by household and community factors, and can be addressed to some extent, but not entirely, by interventions at either level. Low nutritional status is more likely in children of low socioeconomic status, and children at risk are clustered in dry regions and within community with poor environmental status. Inclusion of household and community factors is needed in addressing the issue of child health in Cameroon, and will assist in designing more effective context-specific intervention programs.

## Competing interests

The author(s) declare that they have no competing interests.

## Authors' contributions

RP contributed to the study design, the data analysis and the writing of the manuscript. ME and JAS supervised all parts of the study and contributed to the methodology and writing of the manuscript. All authors read and approved the final version of the manuscript.

## Pre-publication history

The pre-publication history for this paper can be accessed here:



## Figures and Tables

**Table 1:** Regional distribution of clusters, households and children

	Clusters		Households		Children	
	1991	1998	1991	1998	1991	1998
**Regions**						
Yaounde/Douala	43 (28.8%)	43 (21.2%)	483 (13.6%)	553 (11.8%)	260 (13.2%)	201 (8.9%)
West/Littoral	24 (16.1%)	39 (19.2%)	643 (18.2%)	780 (16.6%)	306 (15.6%)	303 (13.4%)
North-West/South West	19 (12.7%)	39 (19.2%)	535 (15.1%)	923 (19.7%)	288 (14.7%)	401 (17.7%)
Center/South/East	23 (15.4%)	41 (20.2%)	669 (18.9%)	1045 (22.2%)	374 (19.0%)	527 (23.3%)
Adamaoua/North/Far North	40 (26.8%)	41 (20.2%)	1208 (34.1%)	1395 (29.7%)	738 (37.5%)	828 (36.6%)
**Type of place of residence**						
Yaounde/Douala	43 (28.9%)	43 (20.7%)	483 (13.6%)	553 (11.8%)	260 (13.2%)	201 (8.9%)
Other cities/towns	37 (24.8%)	50 (24.6%)	835 (23.6%)	983 (20.9%)	518 (26.3%)	421 (18.6%)
Rural	69 (46.3%)	110 (54.2%)	2220 (62.7%)	3160 (67.3%)	1188 (60.4%)	1638 (72.4%)
**Total sample size**	149 (100%)	203 (100%)	3538 (100%)	4696 (100%)	1966 (100%)	2260 (100%)

Notes: -Two clusters in the North-West/South-West region were not surveyed in 1991; so the total number of clusters surveyed was 350 for the two periods.

-The sample sizes for households and children under 3 years old reported in the table are weighted sample sizes; anthropometric measures were taken or were consistent for 1587 children in 1991 and 1923 children in 1998, resulting in a total weighted sample size of 3500 children for the two periods. The non-weighted total sample size was 3321 for children.

-Yaounde and Douala are the largest cities in Cameroon and were sampled separately from other regions and other cities and towns that can be considered as intermediate cities.

**Table 2:** Distribution (%) of variables

**Variables**	**1991**	**1998**	**Variables**	**1991**	**1998**
**N**	1587	1923		1587	1923
**Level 1 variables**			**Level 1 variables (cont'd)**		
**Child characteristics**			**Economic status (ES)**		
**Sex**			1^st ^quintile	16.5	22.6
Male	49.4	50.0	2^nd ^quintile	15.7	23.0
Female	50.6	50.0	3^rd ^quintile	22.6	18.0
**Age (months)**			4^th ^quintile	20.3	19.5
0–5	17.7	19.0	5^th ^quintile	24.9	16.9
6–11	21.1	17.7	*Continuous (Mean)*	*-0.82*	*-0.99*
12–23	34.6	33.9	**MHSB**		
24–35	26.6	29.4	1^st ^quintile	14.8	20.6
**Breastfeeding duration **			2^nd ^quintile	18.3	21.9
Never breastfed	0.9	0.7	3^rd ^quintile	19.5	21.3
Still breastfeeding	56.7	58.9	4^th ^quintile	20.1	20.8
Breastfed 0–4 months	1.9	0.9	5^th ^quintile	27.3	15.4
Breastfed 5–6 months	1.6	0.8	*Continuous (Mean)*	*0.89*	*0.68*
Breastfed 7–18 months	28.5	25.8	**Household environment**		
Breastfed 19 months or more	10.4	12.6	**Water**		
Missing	0.0	0.2	Poor = Well, rain, river	61.2	64.7
**Preceding birth interval**			Medium = Public tap	20.2	17.8
First born child	18.6	22.0	Good = Piped water	18.4	11.2
7–18 months	6.4	4.7	Missing	0.2	6.2
19–23 months	7.8	8.6	**Sanitation**		
24 months or more	67.2	64.7	Poor = Pit latrine	55.3	69.6
**Birth size**			Medium = Bucket or improved pit latrine	7.3	4.0
Smaller than average	15.6	10.8	Good = Flush toilet	37.3	20.4
Average	54.6	52.0	Missing	0.1	6.0
Larger than average	29.8	37.2	**Cooking fuel**		
**Maternal characteristics**			Poor = No electric stove or portable gas	78.9	73.1
**Education**			Good = Electric stove, portable gas	20.9	20.9
No education	34.7	33.5	Missing	0.2	6.0
Primary	41.8	40.2			
Secondary or +	23.5	26.3	**Level 2 variables**		
**Maternal age at birth (yrs)**			**Place of residence**		
<20	22.8	21.1	Yaounde/Douala	14.0	7.7
20–29	51.3	52.5	Intermediate cities	25.7	18.7
30–49	25.8	26.4	Rural	60.3	73.6
**Marital status**			**Region of residence**		
Married monogamy	51.4	52.2	Yaounde/Douala	14.0	7.7
Other	48.6	47.8	West/Littoral	17.6	12.5
**Household characteristics**			North-West/South-West	15.8	17.6
**Indicators for ES (% yes)**			Center/South/East	20.7	24.5
Radio	66.1	52.6	Adamaoua/North/Far-North	31.9	37.7
Electricity	35.3	34.2	**Community environmental status (Mean) **	-0.46	-0.75
Television	22.8	18.3			
Car	7.9	4.7			
**Indicators for MHSB (% yes)**					
Prenatal attendance	83.9	79.5			
Tetanus injection during pregnancy	76.5	70.4			
Medical assistance at delivery	68.0	59.0			
Knowledge of ORS	42.4	56.6			

ABBREVIATIONS: ES = household economic status; MHSB = maternal health-seeking behavior

Notes: The ES and MHSB quintiles were constructed using pooled 1991 and 1998 DHS data to facilitate cross-year comparability. Higher proportion of children in the 1^st ^and 2^nd ^ES and MHSB quintile in 1998 compared with 1991 indicates declines in ES and MHSB during the 1990s, as confirmed by change in the mean value of these variables between the two periods; these changes were statistically significant at 0.001. Also note that ES and MHSB "indicators" were not included as independent variables in multivariate analyses.

**Table 3:** Nutritional status in Cameroon in 1991 and 1998

	1991	1998	P-value of change
	Crude estimates		

WAZ	-0.70	-0.83	0.006
HAZ	-1.03	-1.14	0.026
WHZ	-0.09	-0.16	0.069
% WAZ<-2 SD	16.3	22.2	<0.0001
% HAZ<-2 SD	22.9	29.3	<0.0001
% WHZ<-2 SD	3.8	5.9	0.004
	Standardized estimates^1^		
WAZ	-0.70	-0.79	0.042
HAZ	-1.03	-1.08	0.239
WHZ	-0.09	-0.15	0.138
% WAZ<-2 SD	16.3	20.6	<0.0001
% HAZ<-2 SD	22.8	27.3	0.002
% WHZ<-2 SD	3.8	6.0	0.004

-ABBREVIATIONS: WAZ: weight-for-age z-score; HAZ: height-for-age z-score; WHZ: weight-for-age z-score; % WAZ<-2 SD: percentage of children with weight-for-age z-score 2 standard deviations (SD) below the median value of weight-for-age z-score of the international reference population; the same definition applies to %HAZ<-2SD and %WHZ<-2SD by replacing WAZ by HAZ and WHZ, respectively. ^1 ^The 1998 estimates were standardized by urban/rural place of residence and child sex and age; they measure child nutritional status in 1998 if the distributions of study population across urban and rural areas and across child sex and age in 1998 were similar to the 1991 distributions.

**Table 4:** Average weight-for-age and height-for-age for selected variables

	Weight-for-age z-score			Height-for-age z-score		
	1991	1998	Change	1991	1998	Change

**Household Economic status**						
1^st ^quintile	-1.03	-1.20	-0.17	-1.26	-1.47	-0.21
2^nd ^quintile	-0.71	-0.91	-0.20	-1.02	-1.16	-0.14
3^rd ^quintile	-0.94	-0.96	-0.02	-1.35	-1.27	0.08
4^th ^quintile	-0.51	-0.62	-0.11	-0.95	-0.97	-0.02
5^th ^quintile	-0.42	-0.35	0.07	-0.68	-0.75	-0.07
**MHSB**						
1^st ^quintile	-1.31	-1.34	-0.03	-1.38	-1.54	-0.16
2^nd ^quintile	-0.83	-0.92	-0.09	-1.05	-1.12	-0.07
3^rd ^quintile	-0.66	-0.76	-0.10	-1.11	-1.22	-0.11
4^th ^quintile	-0.64	-0.58	0.06	-0.94	-0.92	0.02
5^th ^quintile	-0.36	-0.47	-0.11	-0.84	-0.84	0
**Household environment**						
**Water**						
Poor	-0.88	-0.94	-0.06	-1.20	-1.23	-0.03
Medium	-0.56	-0.71	-0.15	-0.91	-1.01	-0.10
Good	-0.24	-0.38	-0.14	-0.60	-0.84	-0.24**
**Sanitation**						
Poor	-0.88	-0.94	-0.06	-1.20	-1.24	-0.04
Medium	-0.39	-0.13	0.26	-0.68	-0.51	0.17
Good	-0.50	-0.58	-0.08	-0.84	-0.90	-0.06
**Cooking fuel**						
Poor	-0.25	-0.55	-0.30*	-0.82	-0.88	-0.06
Good	-0.82	-0.94	-0.12**	-1.15	-1.24	-0.09**
**Maternal education**						
No education	-1.08	-1.35	-0.27**	-1.28	-1.48	-0.20
Primary	-0.57	-0.65	-0.08	-1.01	-1.06	-0.05
Secondary or +	-0.38	-0.45	-0.07	-0.71	-0.84	-0.13
**Child sex**						
Male	-0.71	-0.91	-0.20**	-1.03	-1.21	-0.18**
Female	-0.70	-0.76	-0.06	-1.04	-1.08	-0.04
**Child age (months)**						
0–5	0.43	0.45	0.02	-0.02	-0.07	-0.05
6–11	-0.65	-0.80	-0.15	-0.75	-0.64	0.11
12–23	-1.16	-1.35	-0.19**	-1.42	-1.69	-0.27**
24–35	-0.91	-1.09	-0.18**	-1.43	-1.51	-0.08
**Place of residence**						
Yaounde/Douala	-0.16	-0.31	-0.15	-0.57	-0.75	-0.18
Intermediate cities	-0.71	-0.73	-0.02	-1.00	-1.01	-0.01
Rural (reference)	-0.82	-0.91	-0.09	-1.15	-1.22	-0.07
**Region**						
West/Littoral	-0.35	-0.37	-0.02	-0.96	-0.96	0.0
North-West/South-West	-0.45	-0.44	0.01	-1.10	-0.94	0.16
Center/South/East	-0.83	-0.82	0.01	-1.01	-1.12	-0.11
Adamaoua/North/Far-North	-1.17	-1.29	-0.12	-1.25	-1.40	-0.15

-Statistical significance of change: *: P < 0.10; **: p < 0.05; ***: p < 0.001

-Gradient in weight-for-age and height for age z-scores for each independent variable shown in the table was statistically significant at 0.001.

**Table 5:** Multilevel linear regression estimates of the effects of socioeconomic and environmental factors on weight-for-age z-score

	Model 1		Model 2		Model 3		Model 4		Model 5		Model 6		Model 7	
	1991	1998	1991	1998	1991	1998	1991	1998	1991	1998	1991	1998	1991	1998

**Household Economic status**														
1^st ^quintile (reference)														
2^nd ^quintile	0.184	0.174	0.191	0.140			0.252**	0.199**	0.232*	0.222**			0.233*	0.215**
3^rd ^quintile	-0.125	0.129	-0.116	0.121			0.026	0.152	0.003	0.176*			0.006	0.180*
4^th ^quintile	0.194	0.349**	0.181	0.334**			0.160	0.359***	0.071	0.394***			0.064	0.400***
5^th ^quintile	0.320**	0.647***	0.272*	0.638***			0.248*	0.576***	0.049	0.575***			0.028	0.594***
*Continuous*	0.185**	0.290***	0.162**	0.288***			0.104*	0.246***	*0.013*	0.227***			*0.008*	0.227**
**Maternal health-seeking behavior**														
1^st ^quintile (reference)														
2^nd ^quintile	0.352**	0.227**	0.297**	0.047			0.120	-0.050	0.121	-0.038			0.125	-0.071
3^rd ^quintile	0.438**	0.241**	0.349**	-0.087			0.230*	-0.076	0.236*	-0.071			0.229*	-0.143
4^th ^quintile	0.455**	0.331**	0.293*	-0.074			0.203	0.023	0.187	0.036			0.173	-0.059
5^th ^quintile	0.647***	0.359**	0.440**	-0.152			0.355**	0.047	0.308**	0.036			0.279*	-0.079
*Continuous*	0.193***	0.128**	0.125**	*-0.426*			0.137**	*0.043*	0.110**	*0.042*			0.112**	*0.004*
**Household environment**														
**Water**														
Poor (reference)														
Medium					0.167	0.157*			0.040	-0.046			0.006	-0.030
Good					0.470***	0.209*			0.278**	-0.034			0.234**	-0.023
**Sanitation**														
Poor (reference)														
Medium					0.031	0.361**			-0.098	0.212			-0.147	0.195
Good					0.093	0.088			0.052	-0.117			0.042	-0.113
**Cooking fuel**														
Poor (reference)														
Good					0.312**	0.240**			0.218**	0.091			0.188	0.087
**Maternal education**														
No education (reference)														
Primary			0.262**	0.595***							0.133	0.300**	0.055	0.287**
Secondary or +			0.399**	0.732***							0.213*	0.406***	0.091	0.319**
**Maternal age at birth (yrs)**														
<20 (reference)														
20–29							0.032	0.079	0.026	0.084	0.019	0.104	0.026	0.094
30–49							0.017	0.142	0.016	0.135	0.011	0.186*	0.020	0.160
**Maternal marital status**														
Married monogamy							0.182**	0.038	0.175**	0.028	0.186**	0.016	0.234**	0.022
Other (reference)														
**Child sex**														
Male							-0.074	-0.209***	-0.067	-0.208***	-0.072	-0.206***	-0.070	-0.207***
Female (reference)														
**Child age (months)**														
0–5 (reference)														
6–11							-1.121***	-1.222***	-1.124***	-1.237***	-1.114***	-1.210***	-1.126***	-.1.237***
12–23							-1.756***	-1.965***	-1.742***	-1.982***	-1.730***	-1.949***	-1.741***	-1.966***
24–35							-1.646***	-1.899***	-1.637***	-1.913***	-1.617***	-1.869***	-1.637***	-1.895***
Birth size														
Small (reference)														
Average							0.359***	0.188**	0.358***	0.178**	0.379***	0.180**	0.367***	0.175**
Large							0.797***	0.514***	0.796***	0.505***	0.814***	0.497***	0.799***	0.493***
**Breastfeeding duration**														
Never breastfed							-0.240	-0.205	-0.232	-0.110	-0.186	-0.207	-0.209	-0.102
Still breastfeeding							-0.559**	-0.190	-0.490**	-0.128	-0.512**	-0.192	-0.466**	-0.104
Breastfed 0–4 months							-0.067	0.460	-0.036	0.428	-0.035	0.452	-0.020	0.438
Breastfed 5–6 months (reference)														
Breastfed 7–18 months							-0.243	0.232	-0.196	0.288	-0.227	0.208	-0.180	0.297
Breastfed 19 months or more							-0.534**	0.054	-0.465**	0.119	-0.503**	0.061	-0.435*	0.152
**Preceding birth interval**														
First born child							-0.002	0.023	-0.008	0.034	-0.029	0.005	-0.009	0.0250
7–18 months							-0.088	-0.248*	-0.102	-0.233*	-0.099	-0.279**	-0.102	-0.248*
19–23 months							-0.182	-0.864	-0.161	-0.089	-0.164	-0.089	-0.158	-0.096
24 months or more (reference)														
**Place of residence**														
Yaounde/Douala											0.373**	0.366**	0.298	0.337*
Intermediate cities											-0.140	0.001	-0.162	-0.060
Rural														
**Region**														
West/Littoral							0.686***	0.778***	0.663***	0.791***	0.601***	0.533***	0.575***	0.618***
North-West/South-West							0.599***	0.808***	0.605***	0.831***	0.574***	0.558***	0.520***	0.657***
Center/South/East							0.308**	0.356**	0.276**	0.362***	0.211	0.091	0.186	0.177
Adamaoua/North/Far-North (reference)														
**Community environmental index**											0.117**	0.078**	0.074*	0.003
Log-Likelihood	-5729.851		-5705.574		-5739.404		-5198.523		-5183.880		-5205.653		-5177.527	
Number of children	3321		3321		3321		3321		3321		3321		3321	
Number of clusters	350		350		350		350		350		350		350	
**Random-effects parameters**														
Between-individual variance (95 CI)	1.749 (1.663–1.841)		1.745 (1.659–1.836)		1.740 (1.654–1.830)		1.30 (1.232–1.363)		1.285 (1.221–1.352)		1.300 (1.236–1.367)		1.281 (1.218–1.348)	
Between-cluster variance (95 CI)	0.132 (0.089–0.196)		0.095 (0.059–0.153)		0.172 (0.124–0.239)		0.054 (0.031–0.097)		0.053 (0.030–0.097)		0.0572 (0.033–0.099)		0.052 (0.028–0.094)	

Notes: -Each model (except for models 3 and 6) was estimated twice; for the first estimation, economic status and maternal health-seeking behavior were included as continuous variables, all other variables remaining unchanged, and for the second estimation, the quintiles of these variables were included; the table reports coefficients on other variables for the second estimation. A dummy indicator for missing values was also included for each variable with missing values. CI denotes confidence interval.

-Statistical significance: *: P < 0.10; **: p < 0.05; ***: p < 0.001

**Table 6:** Multilevel linear regression estimates of the effects of socioeconomic and environmental factors on height-for-age z-score

	Model 1		Model 2		Model 3		Model 4		Model 5		Model 6		Model 7	
	1991	1998	1991	1998	1991	1998	1991	1998	1991	1998	1991	1998	1991	1998

**Household economic status**														
1^st ^quintile (reference)														
2^nd ^quintile	0.163	0.241**	0.172	0.213*			0.181	0.248**	0.140	0.248**			0.147	0.241**
3^rd ^quintile	-0.186	0.120	-0.171	0.113			-0.115	0.143	-0.152	0.134			-0.141	0.144
4^th ^quintile	0.154	0.312**	0.147	0.298**			0.107	0.300**	-0.023	0.277**			-0.025	0.305**
5^th ^quintile	0.429**	0.560***	0.384**	0.550***			0.317**	0.502***	0.067	0.439**			0.047	0.478**
*Continuous*	0.250***	0.245***	0.221***	0.239***			0.146**	0.206**	*0.011*	0.160**			*-0.007*	0.176**
**Maternal health-seeking behavior**														
1^st ^quintile (reference)														
2^nd ^quintile	0.275*	0.295**	0.217	0.160			0.146	0.129	0.167	0.124			0.175	0.102
3^rd ^quintile	0.147	0.135	0.054	-0.111			0.117	-0.037	0.128	-0.035			0.117	-0.089
4^th ^quintile	0.301*	0.361**	0.132	0.054			0.234	0.253*	0.214	0.248*			0.193	0.167
5^th ^quintile	0.326**	0.365**	0.112	-0.045			0.259	0.260*	0.204	0.237			0.155	0.117
*Continuous*	0.087*	0.123**	*0.06*	*-0.007*			*0.083*	0.108**	*0.060*	0.103**			*0.050*	*0.071*
**Household environment**														
**Water**														
Poor (reference)														
Medium					0.189*	0.146			0.119	-0.026			0.073	0.014
Good					0.381**	0.090			0.254**	-0.134			0.190	-0.094
**Sanitation**														
Poor (reference)														
Medium					0.045	0.496**			-0.080	0.399**			-0.150	0.402**
Good					0.125	0.219**			0.089	0.042			0.076	0.051
**Cooking fuel**														
Poor (reference)														
Good					0.380***	0.187**			0.292**	0.059			0.245**	0.064
**Maternal education**														
No education (reference)														
Primary			0.206*	0.356***							0.141	0.258**	0.100	0.199
Secondary or +			0.374**	0.535***							0.241*	0.459***	0.150	0.313**
**Maternal age at birth (yrs)**														
<20 (reference)														
20–29							0.015	0.100	-0.004	0.098	0.012	0.129	-0.001	0.106
30–49							0.044	0.137	0.040	0.131	0.047	0.188*	0.051	0.157
**Maternal marital status**														
Married monogamy							0.223**	-0.022	0.212**	-0.029	0.230**	-0.032	0.222**	-0.030
Other (reference)														
**Child sex**														
Male							-0.065	-0.198**	-0.052	-0.198**	-0.065	-0.196**	-0.056	-0.198**
Female (reference)														
**Child age (months)**														
0–5 (reference)														
6–11							-0.727***	-0.612***	-0.741***	-0.620***	-0.718***	-0.589***	-0.745***	-0.614***
12–23							-1.511***	-1.770***	-1.497***	-1.769***	-1.492***	-1.745***	-1.497***	-1.752***
24–35							-1.664***	-1.743***	-1.653***	-1.743***	-1.645***	-1.707***	-1.656***	-1.727***
Birth size														
Small (reference)														
Average							0.302**	0.120	0.315**	0.124	0.325**	0.114	0.329**	0.124
Large							0.552***	0.352***	0.567***	0.124***	0.566***	0.356***	0.572***	0.351***
**Breastfeeding duration**														
Never breastfed							-0.258	-0.687	-0.258	-0.691	-0.190	-0.679	-0.221	-0.652
Still breastfeeding							-0.407*	-0.412	-0.335	-0.343	-0.313	-0.410	-0.297	-0.307
Breastfed 0–4 months							0.052	-0.108	0.081	-0.969	0.104	-0.100	0.110	-0.076
Breastfed 5–6 months (reference)														
Breastfed 7–18 months							-0.027	-0.103	0.019	-0.041	0.036	-0.111	0.046	-0.013
Breastfed 19 months or more							-0.213	-0.189	-0.144	-0.123	-0.142	-0.179	-0.095	-0.077
**Preceding birth interval**														
First born child							0.032	-0.074	0.029	-0.075	0.022	-0.100	0.030	-0.088
7–18 months							-0.021	-0.233	-0.036	-0.237	-0.023	-0.274*	-0.033	-0.253*
19–23 months							-0.152	-0.224**	-0.129	-0.221**	-0.130	-0.220*	-0.125	-0.227**
24 months or more (reference)														
**Place of residence**														
Yaounde/Douala											-0.092	0.276	-0.198	0.175
Intermediate cities											-0.206*	0.158	-0.245*	0.041
Rural (reference)														
**Region**														
West/Littoral							0.218	0.293**	0.185	0.273**	0.069	0.152	0.052	0.160
North-West/South-West							0.143	0.400**	0.150	0.406**	0.028	0.268**	0.025	0.315**
Center/South/East							0.251*	0.175	0.217*	0.161	0.096	-0.013	0.072	0.024
Adamaoua/North/Far-North (reference)														
**Community environmental index**											0.156***	0.021	0.103**	-0.049
Log-Likelihood	-5951.931		-5939.119		-5953.087		-5594.023		-5578.431		-5600.445		-5571.327	
Number of children	3321		3321		3321		3321		3321		3321		3321	
Number of clusters	350		350		350		350		350		350		350	
**Random-effects parameters**														
Between individual variance (95 CI)	2.059 (1.958–2.165		2.055 (1.954–2.161)		2.057 (1.957–2.163)		1.660 (1.578–1.746)		1.649 (1.567–1.734)		1.675 (1.592–1.762)		1.647 (1.565–1.732)	
Between cluster variance (95 CI)	0.059 (0.029–0.118)		0.042 (0.017–0.105)		0.063 (0.033–0.121)		0.047 (0.022–0.100)		0.041 (0.018–0.095)		0.036 (0.015–0.090)		0.034 (0.013–0.090)	

Notes: -Each model (except for models 3 and 6) was estimated twice; for the first estimation, economic status and maternal health-seeking behavior were included as continuous variables, all other variables remaining unchanged, and for the second estimation, the quintiles of these variables were included; the table reports coefficients on other variables for the second estimation. A dummy indicator for missing values was also included for each variable with missing values. CI denotes confidence interval.

-Statistical significance: *: P < 0.10; **: p < 0.05; ***: p < 0.001

**Table 7:** Change in the coefficients of selected variables between 1991 and 1998

	Weight-for-age z-score		Height-for-age z-score	
	Unadjusted model	Adjusted model	Unadjusted model	Adjusted model

**Household Economic status**				
1^st ^quintile (reference)				
2^nd ^quintile	-0.027	-0.017 (-0.009)	0.081	0.094 (0.107)
3^rd ^quintile	0.194	0.174 (0.172)	0.302*	0.286* (0.286*)
4^th ^quintile	0.087	0.337* (0.322*)	0.179	0.331* (0.301)
5^th ^quintile	0.238	0.566** (0.526**)	0.164	0.431* (0.371*)
*Continuous*	*0.055*	0.219** (0.214**)	*-0.017*	*0.183 (0.150)*
**MHSB**				
1^st ^quintile (reference)				
2^nd ^quintile	-0.086	-0.196 (-0.160)	0.057	-0.073 (-0.043)
3^rd ^quintile	-0.144	-0.372* (-0.308*)	0.007	-0.206 (-0.163)
4^th ^quintile	-0.044	-0.232 (-0.150)	0.059	-0.026 (0.034)
5^th ^quintile	-0.142	-0.358* (-0.272)	0.106	-0.038 (0.033)
*Continuous*	*-0.039*	*-0.108 (-0.068)*	*0.022*	*0.021 (0.043)*
**Household environment**				
**Water**				
Poor (reference)				
Medium	-0.018	-0.036 (-0.087)	-0.042	-0.059 (-0.145)
Good	-0.215	-0.257 (-0.312**)	-0.250*	-0.284 (-0.387**)
**Sanitation**				
Poor (reference)				
Medium	0.552***	0.342 (0.310)	0.078	0.553** (0.479**)
Good	-0.084	-0.155 (-0.169)	-0.031	-0.024 (-0.047)
**Cooking fuel**				
Poor (reference)				
Good	-0.114	-0.101 (-0.127)	-0.201*	-0.180 (-0.233*)
**Maternal education**				
No education (reference)				
Primary	0.142	0.232 (0.167)	0.066	0.099 (0.117)
Secondary or +	0.159	0.229 (0.193)	0.074	0.162 (0.218)
**Maternal marital status**				
Married monogamy	-0.223**	-0.212* (-0.147*)	-0.295**	-0.251** (-0.241**)
Other (reference)				
**Child sex**				
Male	-0.187**	-0.137* (-0.141*)	-0.154	-0.142 (-0.145)
Female (reference)				
**Child age (months)**				
0–5 (reference)				
6–11	-0.129	-0.111 (-0.113)	0.089	0.130 (0.120)
12–23	-0.216*	-0.225* (-0.240*)	-0.320**	-0.255* (-0.271*)
24–35	-0.204	-0.257 (-0.275)	-0.146	-0.071 (-0.090)
**Place of residence**				
Yaounde/Douala	-0.160	0.039 (-0.007)	-0.139	0.373 (0.368)
Intermediate cities	0.071	0.102 (0.141)	0.084	0.286 (0.364**)
Rural (reference)				
**Region**				
West/Littoral	-0.125	0.043 (-0.068)	0.091	0.107 (0.082)
North-West/South-West	0.107	0.137 (-0.016)	0.237	0.291 (0.240)
Center/South/East	0.053	-0.008 (-0.120)	0.003	-0.048 (-0.109)
Adamaoua/North/Far-North (reference)				
**Community environmental index**	-0.032	-0.070 (-0.040)	-0.049	-0.152** (-0.134**)

Notes: The unadjusted models included only the independent variable of interest; the adjusted models additionally included all other independent variables used in this study (see tables 5–6, model 7); values in parentheses were estimated from model 5 for all variables in the table except for maternal education, place of residence, region and community environmental index which were estimated from model 6. Only selected variables are presented in the table.

-Statistical significance: *: P < 0.10; **: p < 0.05; ***: p < 0.001

**Table 8:** Age-specific effects of household economic status (ES)

	Weight-for-age z-score				Height-for-age z-score			
	1991		1998		1991		1998	

Child age groups (months)	Unadjusted model	Adjusted model	Unadjusted model	Adjusted model	Unadjusted model	Adjusted model	Unadjusted model	Adjusted model

	**Effects of ES**							
0–5	0.095	-0.153	0.055	-0.047	-0.069	-0.275**	0.313**	0.202*
6–11	0.331***	0.127	0.427***	0.295**	0.260**	0.087	0.194*	0.062
12–23	0.404***	0.176**	0.503***	0.361***	0.473***	0.268**	0.408***	0.269**
24–35	0.399***	0.177**	0.445***	0.306***	0.510***	0.332***	0.381***	0.225**
	**Age-difference in ES effects (each age group is compared to 0–5 months)**							
0–5 (reference)								
6–11	0.236*	0.280**	0.371**	0.341**	0.329**	0.363**	-0.118	-0.140
12–23	0.308**	0.330**	0.448***	0.407***	0.542***	0.543***	0.094	0.067
24–35	0.304**	0.330**	0.389***	0.353***	0.579***	0.608***	0.068	0.023

Notes: The unadjusted models for "ES effects" included child age groups and the interaction terms between child age groups and ES; the adjusted models additionally included the independent variables used in model 4 of tables 5–6 (model 4 was used to avoid correlation of ES with variables such as education and community environmental index; however the full model (model 7) yielded results very similar to those presented in the table). The unadjusted models for "age-difference in ES effects" included child age groups, ES, and the interaction terms between child age groups and ES (except for the interaction term between 0–5 months and ES); the adjusted models additionally included the independent variables used in model 4 of tables 5–6. These models were estimated for the years 1991 and 1998 simultaneously.

-Statistical significance: *: P < 0.10; **: p < 0.05; ***: p < 0.001
